# Broadband and Highly Directional Visible Light Scattering by Laser-Splashed Lossless TiO_2_ Nanoparticles

**DOI:** 10.3390/molecules26206106

**Published:** 2021-10-10

**Authors:** Yinan Zhang, Shiren Chen, Jing Han

**Affiliations:** 1 Institute of Photonic Chips, University of Shanghai for Science and Technology, Shanghai 200093, China; 2 Centre for Artificial-Intelligence Nanophotonics, School of Optical-Electrical and Computer Engineering, University of Shanghai for Science and Technology, Shanghai 200093, China; 3 Institute of Photonics Technology, Jinan University, Guangzhou 510632, China; chen.shiren@foxmail.com (S.C.); hanjing@stu2018.jnu.edu.cn (J.H.)

**Keywords:** directional scattering, dielectric nanoparticles, laser fabrication

## Abstract

All-dielectric nanoparticles, as the counterpart of metallic nanostructures have recently attracted significant interest in manipulating light-matter interaction at a nanoscale. Directional scattering, as an important property of nanoparticles, has been investigated in traditional high refractive index materials, such as silicon, germanium and gallium arsenide in a narrow band range. Here in this paper, we demonstrate that a broadband forward scattering across the entire visible range can be achieved by the low loss TiO_2_ nanoparticles with moderate refractive index. This mainly stems from the optical interferences between the broadband electric dipole and the magnetic dipole modes. The forward/backward scattering ratio reaches maximum value at the wavelengths satisfying the first Kerker’s condition. Experimentally, the femtosecond pulsed laser was employed to splash different-sized nanoparticles from a thin TiO_2_ film deposited on the glass substrate. Single particle scattering measurement in both the forward and backward direction was performed by a homemade confocal microscopic system, demonstrating the broadband forward scattering feature. Our research holds great promise for many applications such as light harvesting, photodetection and on-chip photonic devices and so on.

## 1. Introduction

As the building block of metamaterials and metasurfaces, nanoparticles play a crucial role in controlling light propagation and regulating the light field distribution [1,2]. Although metal nanoparticles supporting localized surface plasmons have been widely investigated and are found in many applications such as Raman scattering enhancement [3], biosensing [4], metasurfaces [5], nonlinear plasmonics [6,7] and photovoltaics [8,9,10], their large optical losses and lack of magnetic resonances have hindered their further development. All-dielectric nanomaterials have recently emerged as a promising alternative to plasmonic nanoparticles in constituting high-efficiency photonic devices due to the coexistence of electric and magnetic resonances and lower optical losses as well. 

Directional scattering is an important property in nanoparticles that can be used to make Huygens directional sources [11] and enhance solar energy harvesting [12,13] and photodetector responses. Previous studies have demonstrated that directional scattering is the result of amplitude and phase superposition due to interferences between different modes in the nanoparticles [14,15]. The research is mainly focused on high refractive index materials such as silicon [16], gallium nitride [17] and germanium [18]. Among them, silicon is the most widely used material due to its earth abundance and mature processing technologies. Andrey et al. reported the spattering of silicon particles by femtosecond laser ablation and observed the strong magnetic response and high-order dipole resonance [15]. Following this, Yuan Hsing Fu et al. observed the directional scattering behavior of silicon nanoparticles in the visible light range [19]. Later on, a series of work has been carried out to improve the directional scattering efficiency and its operation wavelengths by other mechanisms, such as the Fano resonance [18,20] and the multibody enhanced electric field [21].

However, the high refractive index nanoparticles such as Si always have their electric dipole and magnetic dipole resonances separated from each other in the broad spectrum, and this leads to rather narrow band directional scattering. Although the non-negligible imaginary part of the high refractive index nanoparticles, such as Ge, can red- and blue-shift the electric and magnetic dipole resonances and make them closer, the large optical loss inevitably limits the directivity efficiency. With the refractive index reduced, the magnetic dipole resonance is shifted closer to the electric dipole modes and can potentially enhance the optical mode interaction and the corresponding directionality. It has been experimentally demonstrated that forwardly directional scattering behavior exists in copper oxide [22]. However, the relatively large optical loss derived from the non-negligible imaginary part of the refractive index of copper oxide and the large scattering strength difference between electric and magnetic dipole resonance empower their forward/backward scattering ratio at a low level. 

Here, we proposed TiO_2_ nanoparticles for directing light scattering with a moderate real refractive index of ~2.4 and an imaginary part of zero in the visible range [23,24]. Through finite difference time domain (FDTD) calculation and Mie scattering theory [25,26] analysis, we find that in the entire visible band, the forward scattering dominates with the forward/backward (F/B) scattering, with large tolerance on the particle morphology, reaching maximum value at the wavelength and satisfying the first Kerker’s condition. Experimentally, we prepared TiO_2_ nanoparticles by a simple femtosecond laser ablation method [16,27,28]. Nanoparticles with diameters between 200 nm and 260 nm were successfully obtained on ITO substrates. Forward and backward scattering spectra were measured by a homemade dark-field scattering microscope attached to a spectrometer. The experimental results are in good agreement with the simulations, with the forward scattering dominating across the entire visible band.

## 2. Results and Discussions

**Figure 1:**
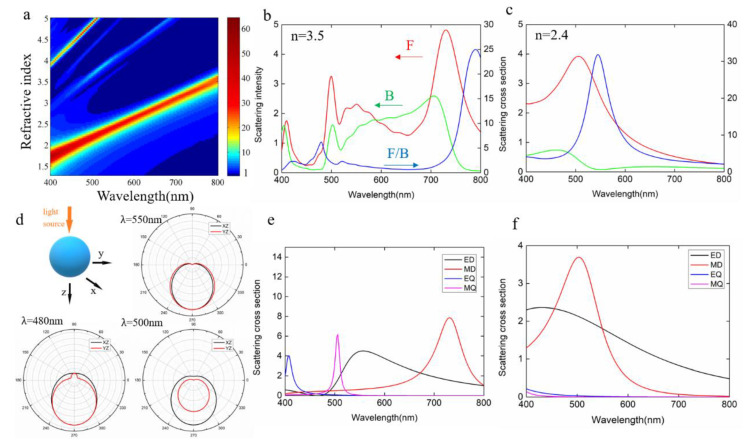
Effect of the refractive index on the forward/backward scattering. (**a**) The F/B value as a function of wavelength for spherical nanoparticles with a refractive index from 1.3 to 5. (**b**,**c**) Forward and backward scattering and F/B spectra for spherical nanoparticles with a refractive index of 3.5 and 2.4. (**d**) Far-field scattering patterns of TiO_2_ nanoparticles at the wavelengths of 480 nm, 500 nm and 550 nm. (**e**,**f**) Multipolar decomposition by Mie scattering theory.

Since the refractive index of the nanoparticles has noticeable effect on the light scattering behavior, we first performed a refractive index search for the directionality features of the nanoparticles, with the results shown in Figure 1a, where the F/B scattering ratio of a spherical nanoparticle with 200 nm diameter located in the air is shown. In this simulation, the imaginary part of the refractive index was set as zero to solely investigate the effect of the magnitude of the real part. A total-field scattered field light source was employed, and the boundary conditions were set as perfectly matched layers. The mesh size of the simulation region was 2 nm, ensuring high accuracy simulation results. We can clearly observe a unique refractive index region (2 < n < 2.7), where the forward scattering dominates in the entire visible range, with distinct peaks for the F/B scattering ratio. When the refractive index is larger than 2.7, significant backward scattering starts to occur and light directionality in both forward and backward scattering occurs in the visible band. Indeed, previous reports have shown that high refractive index materials such as Si and Ge can introduce stronger scattering at both the forward and backward direction, dependent on the wavelengths. However, so far, very few research studies on the nanoparticle with moderate refractive index have been reported. Figure 1b,c compare the scattering directionalities of the nanoparticles with a refractive index of 3.5 and 2.4, representing high and moderate refractive index materials. Clearly, for the high refractive index 3.5, in the short wavelength range below 600 nm and long wavelength range above 700 nm, the forward scattering outperforms backward scattering while in the range between 600 and 700 nm, the backward scattering dominates. On the other hand, for the nanoparticles with a refractive index of 2.4, forward scattering is significantly larger than backward scattering in the entire visible range, with a maximum F/B ratio >30 at around 550 nm. Since the directionalities stem from the multipolar interferences in the nanoparticles, we performed multipolar decomposition for the corresponding nanoparticle, with the results shown in Figure 1d,e, respectively. For high refractive index nanoparticles, ED and MD are separated from each other, and high-order modes EQ and MQ appear in the short band. The first and second Kerker’s conditions are satisfied at around 800 nm and 680 nm, leading to the dominating forward and backward scattering, respectively, at these two wavelengths. As the refractive index decreases to 2.4, ED and MD begin to overlap with each other and constructive interference occurs across the entire band, leading to an enhanced forward scattering. Figure 1d shows the far-field scattering patterns of a 200 nm TiO_2_ particle at three different wavelengths, corresponding to the positions of forward (500 nm) and backward (480 nm) scattering peaks and F/B peak (550 nm). It can be found that at these four different wavelengths, the TiO_2_ particles have significant forward directivity. Unlike high refractive index materials Si or Ge, TiO_2_ maintains good directionality beyond the F/B peak. This demonstrates that the moderate refractive index materials not only possess lower loss, but also show better directionality. Compared to the use of Fano resonance or multipolymer structure to enhance and regulate the directional behavior, moderate refractive index media only need to consider their own size and operating band.

**Figure 2:**
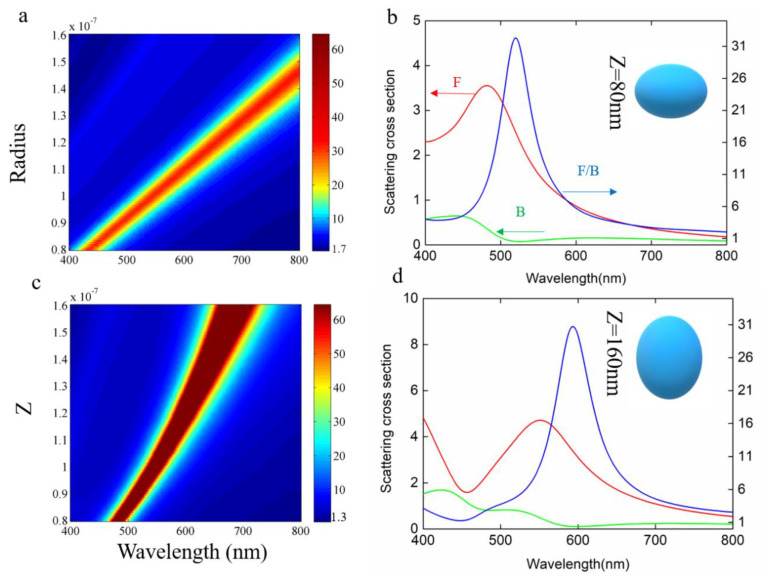
Shape effect of TiO_2_ nanoparticles on scattering. (**a**) The effect of the radius on the F/B value with the color representing the F/B value. (**b**) The effect of the Z on the F/B value, with the color representing the F/B value. (**c**,**d**) Forward scatter and backscatter with the same radius, different Z sizes and F/B values.

The size of nanoparticles has a significant influence on the scattering properties. Therefore, we calculated the F/B value of spherical TiO_2_ nanoparticles with a radius from 80 nm to 150 nm, with the results shown in Figure 2a. It can be seen that as the size increases, the F/B peak value gradually red-shifts over the entire visible spectral range. The F/B value is always larger than one, demonstrating their broadband forward scattering feature. Considering the experimental fabricated nanoparticles are not always ideally spherically shaped, we simulated ellipsoidal-shaped nanoparticles with varied height R_z_ (40–150 nm) for a fixed R_x_ and R_y_ value of 100 nm. As the value of R_z_ increases, the F/B peak gradually red-shifts but the value is still larger than 1 through the entire visible spectral range. Therefore, TiO_2_ is a kind of forwardly directional scattering nanomaterial with large shape tolerance, favorable for stringent nanofabrication processes.

**Figure 3:**
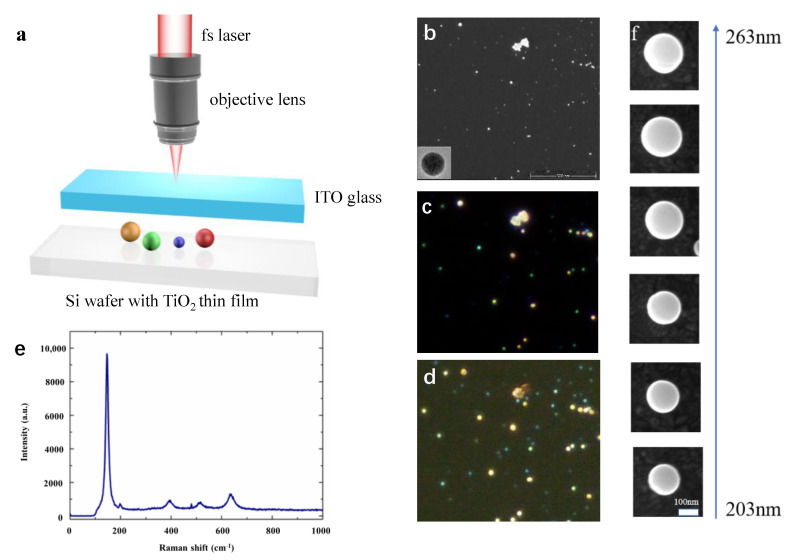
(**a**) Schematic of the laser-splashed process for nanoparticle fabrication. (**b**) SEM image of fabricated nanoparticles with the inset showing the TEM image of one representative nanoparticle. (**c**,**d**) Reflective and transmissive dark-field microscopy images of the areas corresponding to the SEM image area. (**e**) The Raman spectrum of one representative TiO_2_ nanoparticle. (**f**) SEM images of selected nanoparticles with various diameters from 203 nm to 263 nm.

In order to verify the scattering directionality of TiO_2_ nanoparticles, we used the fs pulsed laser ablation method to prepare TiO_2_ nanoparticles, similar to that used in [12]. Figure 3a is the schematic diagram of the process by which TiO_2_ nanoparticles were obtained. Thin film with a thickness of about 200 nm was deposited on a silicon wafer. Then an fs laser (Coherent, Libra) with a central wavelength of 800 nm and a frequency of 1000 HZ was tightly focused on the TiO_2_ thin film surface by an objective lens with an NA = 0.7. A point-by-point method was used to scan the sample. The exposure time was set as 100 ms and the average laser power was 0.5 mW. Due to the local temperature instant rise by the high-intensity energy of fs pulses, a large quantity of nanoparticles was splashed and the ITO glass on top of the Si wafer with a certain distance was used to collect the spattered nanoparticles. ITO glass instead of normal glass was used for the purpose of imaging the nanoparticles using a conductive substrate in the scanning electron microscopy measurement.

Figure 3b shows the SEM image of a selected area of the ITO glass distributed with varied sizes of nanoparticles. The lower left corner is the TEM image of an individual particle. The corresponding dark-field images in both forward and backward directions are shown in Figure 3c,d, respectively. Figure 3e is the Raman spectrum of the TiO_2_ nanoparticles that we made, demonstrating that the fs laser does not change the inherent properties of the TiO_2_ thin film. It can be seen from Figure 3c that the different particle colors cover the entire visible light region from blue to red as the size increases. From Figure 3c we can see that, unlike backward scattering, the color of the particles in the forward scattering dark-field image is red-shifted as a whole, and many particles become white-like colors. For this, we selected six different-sized particles (Figure 3f) with a diameter that gradually increased from 203 nm to 263 nm. We used a self-made microscope system and spectrometer to collect the forward and back spectra of these nanoparticles and compared their forward/backward scattering. 

**Figure 4:**
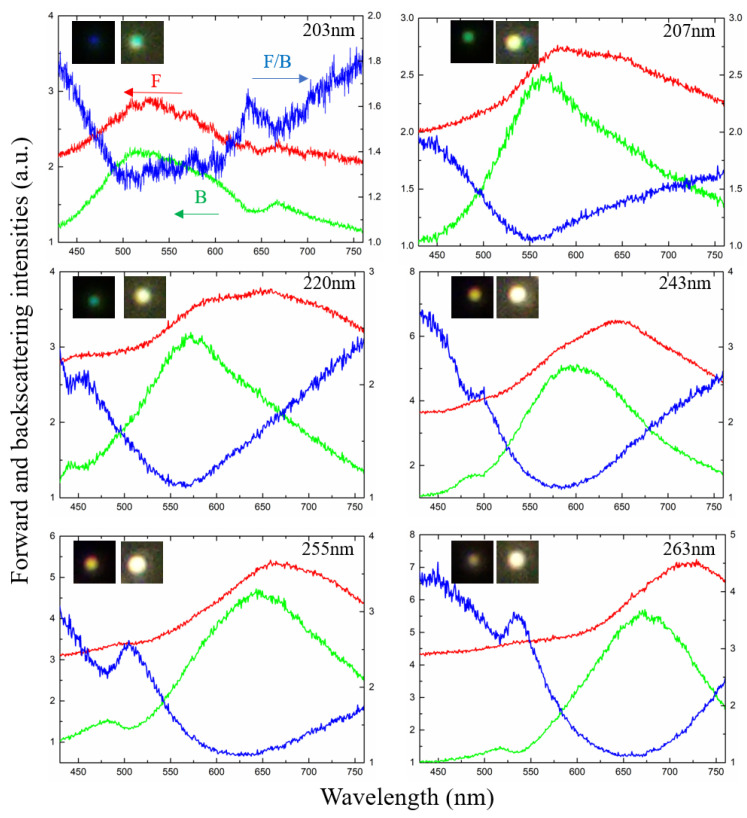
Experimentally measured forward and backward scattering spectra of six selected sizes of nanoparticles. Left axes show forward (red) and backward (green) scattering intensities, and right axes show F/B ratio (blue) of nanoparticles.

In order to obtain the forward/backward scattering ratio of the particles we obtained, we further performed spectral measurements on the selected six particles. Figure 4 presents the forward and backward scattering from the selected six individual nanoparticles and the forward/backward scattering ratio. The forward/backward scattering was normalized to the scattering by a subwavelength dust spot. The inset images in the figures are their reflection dark-field microscope images and transmission dark-field images, respectively, corresponding to backward scattering and forward scattering. It can be seen from Figure 4 that as the size increases, the backward scattering peak gradually red-shifts from ~500 nm to ~650 nm, with the accompanying color appearance of the particles changing from blue to purple. The forward scattering curve also red-shifts as the size increases, with the peak value longer than that of the backward scattering, agreeing well with the simulation results. Furthermore, the spectra in forward scattering mostly locate at the red region with a broad-spectrum distribution, corresponding to the yellow-white colors in the dark-field images.

From the F/B values of different-sized nanoparticles measured in Figure 4, we can see TiO_2_ nanoparticles can achieve F/B > 1 across the entire visible band between 400 nm and 800 nm. The F/B peak gradually red-shifts as the size increases. It is noted that experimental F/B is relatively small, with a maximum value of ~4.5. This is probably because the transmitted light source passes through an attenuator and is incident on the ITO glass with a certain absorption, which leads to a decrease in relative intensity and a slight shift in peak value. Unlike the simulation, the experimentally measured F/B value also has an ascending process in the range of 650–750 nm, which is related to the chromatic aberration that may be caused by the lens combination during absorption and measurement of the ITO material at different wavelengths. Nevertheless, the basic trend of broadband forward scattering domination in the visible range is still consistent with the predictions from simulations. 

## 3. Conclusions

In summary, we have numerically calculated the forward/backward scattering of TiO_2_ nanoparticles and their ratio in the entire visible region. Anisotropic scattering with the forward part dominating was found in the entire visible range with large tolerance on the particle shape and size. This is owing to the multipolar interference between the broadband ED and MD modes in the TiO_2_ nanoparticles. It is different from the polymer-enhanced electric field and the Fano resonance between the particles to enhance and regulate the F/B. Meanwhile, the imaginary part of TiO_2_ nanoparticles in the visible light band is zero, so the optical loss is extremely low. Experimentally, we prepared different sizes of TiO_2_ nanoparticles by the laser ablation method and measured the forward and backward scattering spectra using a homemade microscope system to verify our calculations. Our work has contributed to the determination of directional scattering conditions in the full visible band, and confirmed the broadband directional scattering properties of lossless TiO_2_ nanoparticles.

## Data Availability

Not applicable.
